# Supplementary material for: AURKB as a target in non-small cell lung cancer with acquired resistance to anti-EGFR therapy
Source: Nat Commun. 2019 Apr 18;10:1812. doi: 10.1038/s41467-019-09734-5 (PMC6472415; doi:10.1038/s41467-019-09734-5)
Supplement: Supplementary file 1 — Supplementary Information [file 41467_2019_9734_MOESM1_ESM.pdf]

AURKB as a target in non-small cell lung cancer with acquired resistance to anti-EGFR therapy

Bertran-Alamillo et al.

SUPPLEMENTARY FIGURES

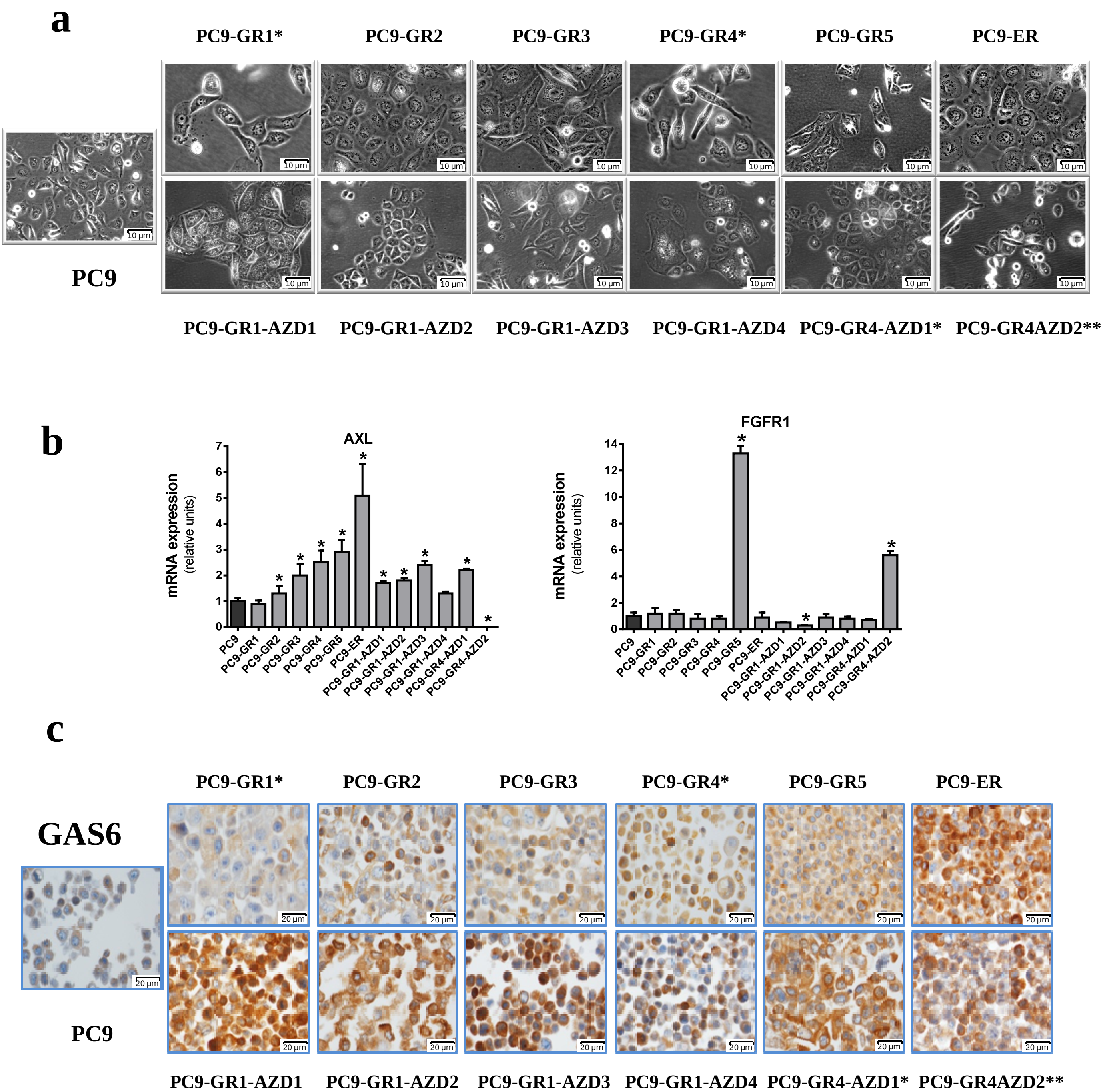

Supplementary Fig. 1: Characterization of the EGFR TKI resistant cell lines used in this study. **a** Microscopic photographs of the parental and resistant PC9 cells. Asterisks indicated cell lines harboring the T790M resistant mutation at >0.1% (*) or <0.1% (**) allelic fraction. Scale bars indicate 10 µm**. b** Relative expression levels of the *AXL* and *FGFR* mRNAs. No significant differences were found on the case of *MER* and *MET*. Experiments were conducted in triplicates, values shown are means ± S.D. and asterisks indicate statistical significance (*P*<0.05 in a Student's t test). **c** IHC staining of GAS6, showing the cytoplasmic localization of the protein in all cell lines of the panel. Images were obtained with an x40 objective. Scale bars indicate 20 µm**.**

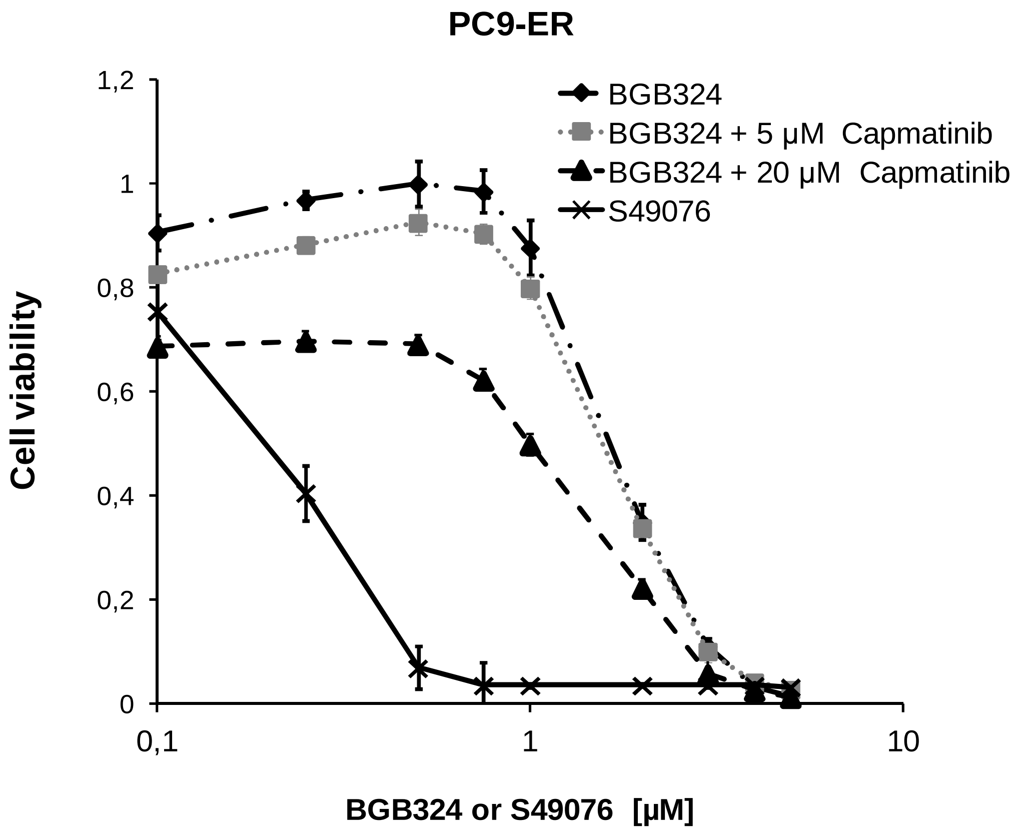

Supplementary Fig. 2: Dose-response plots of PC9-ER cells treated with BGB324, BGB324 in combination with capmatinib and S49076. Experiments were conducted in triplicates, values shown are means ± S.D.

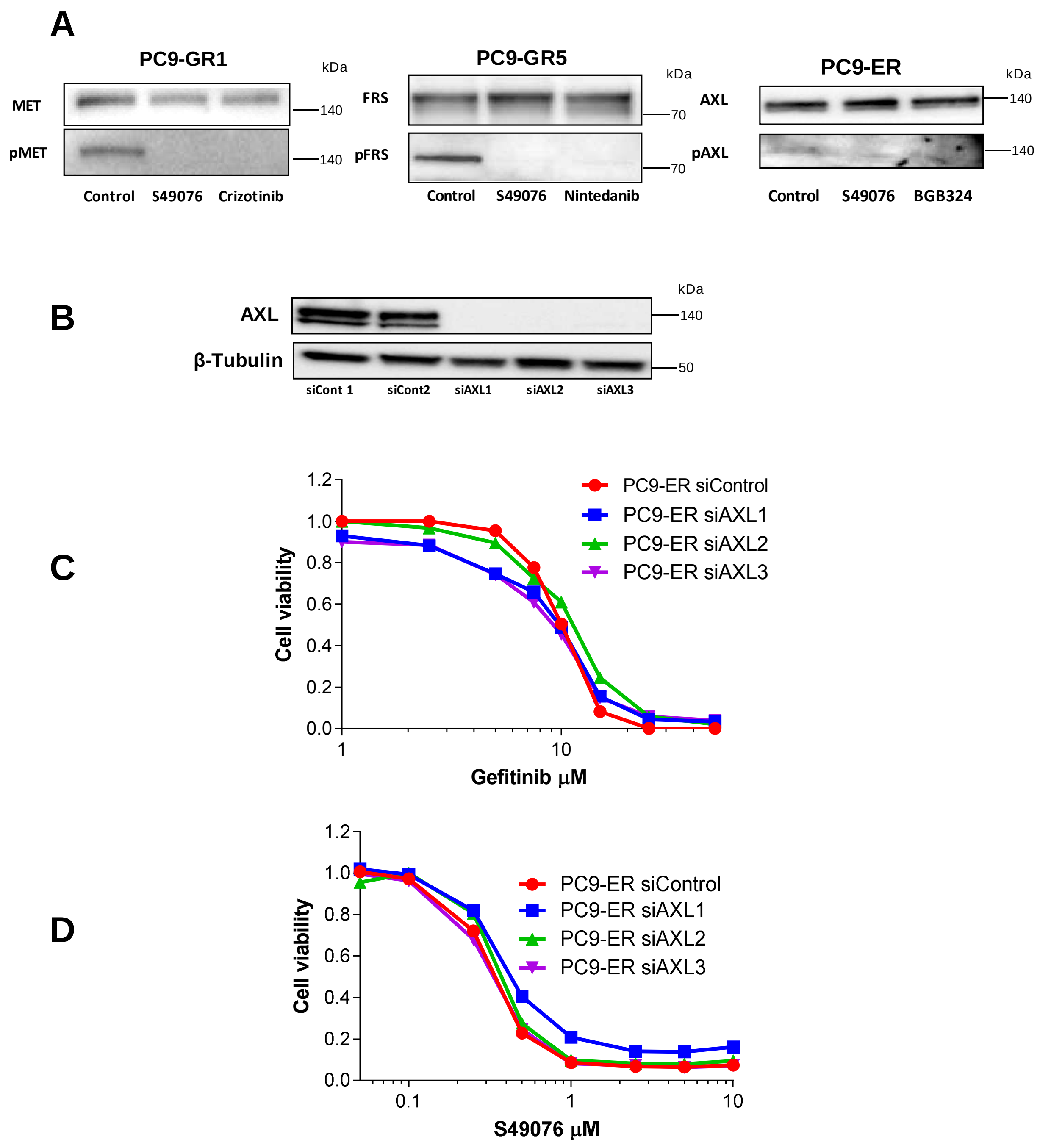

Supplementary Fig. 3: a Effects of S49076, crizotinib and nintedanib and BGB324 on the phosphorylation of MET, FRS and AXL. **b** Expression of AXL by Western blotting in the control and the silenced clones. **c, d** Dose-response plots to gefitinib and S49076 of the PC9-ER control clones and the clones with stable *AXL* silencing. Experiments were conducted in triplicates, values shown are means

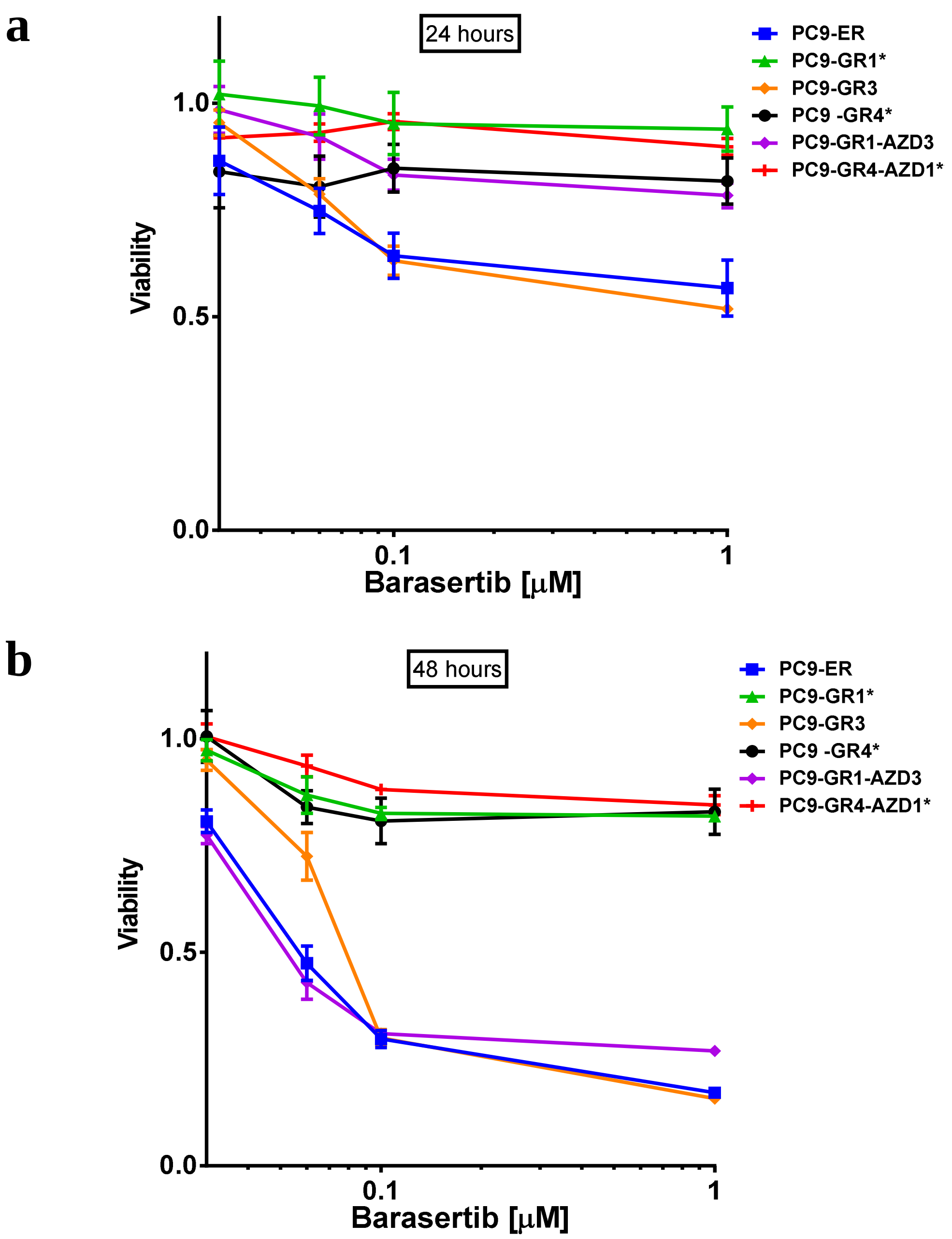

Supplementary Fig. 4: Dose-response curves of selected parental and EGFR TKI resistant cells to the AURKB inhibitor barasertib at 24 (**a**) and 48 h (**b**). Asterisks indicated cell lines harboring the T790M resistant mutation. Experiments were conducted in triplicates, values shown are means ± S.D.

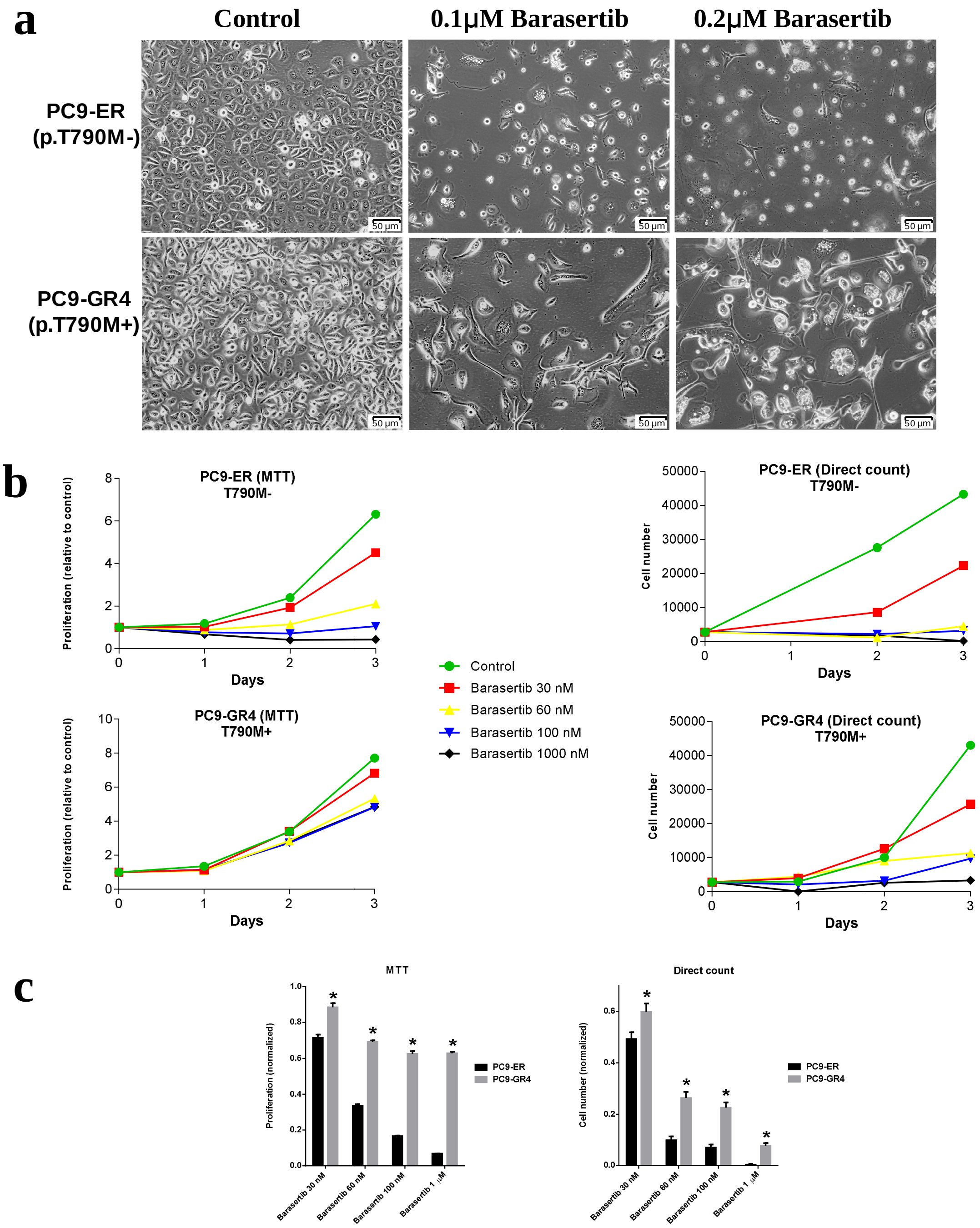

Supplementary Fig. 5: Effects of Barasertib on PC9-ER (p.T790M-negative) and PC9-GR4 (p.T790M-positive) cell lines. **a** Microscopic photographs of the cells at different concentrations of the inhibitor. Scale bars indicate 50 µm. **b** Growth curves of the cell lines in presence of the inhibitors, determined by MTT (left) or direct counting (right). **c** Comparison of the final numbers of cells of the growth curves presented in (**b**), as estimated by MTT (left) or direct count (right). Experiments were conducted in triplicates, values shown are means ± S.D. and asterisks indicate statistical significance ($P<0.05$ in a Student's t test)

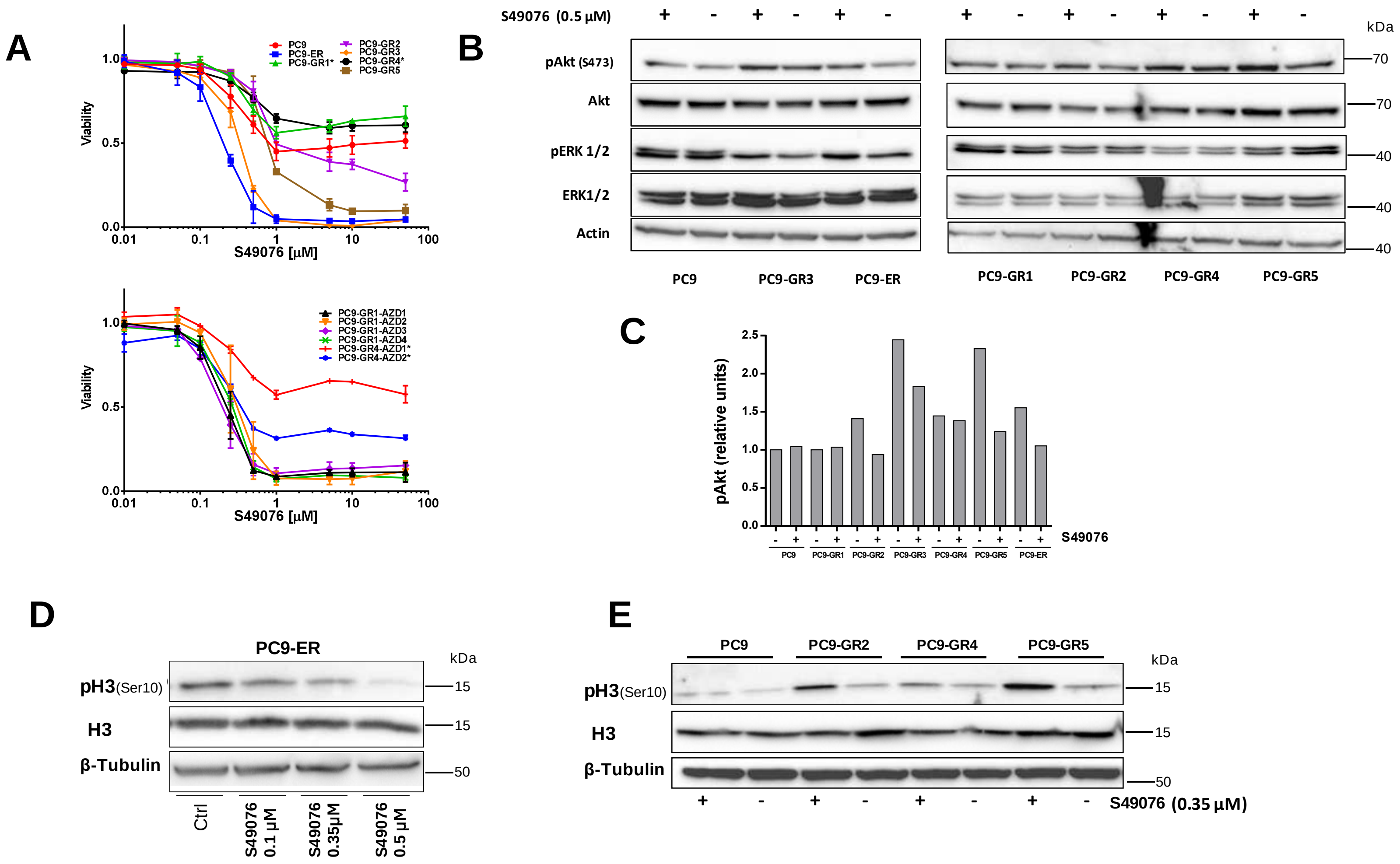

Supplementary Fig. 6: Effects of the MET/AXL/FGFR/AURKB inhibitor S49076 on EGFR TKI resistant cell lines. **a** Dose-response curves of the parental and the EGFR TKI resistant cells to S49076. Experiments were conducted in tri or quadruplicates and values shown are means ± S.D. **b** Effects of the S49076 on the phosphorylation of Erk1/2 and Akt. Incubation time was 2 h. **c** Quantification of the Western blot for pAkt. **d** Western blot showing the dose-dependent decrease of pH3 levels induced by S49076 at 24 h. **e** Western blot demonstrating the inhibition of pH3 by S49076 after 24 h in EGFR-mut cell lines

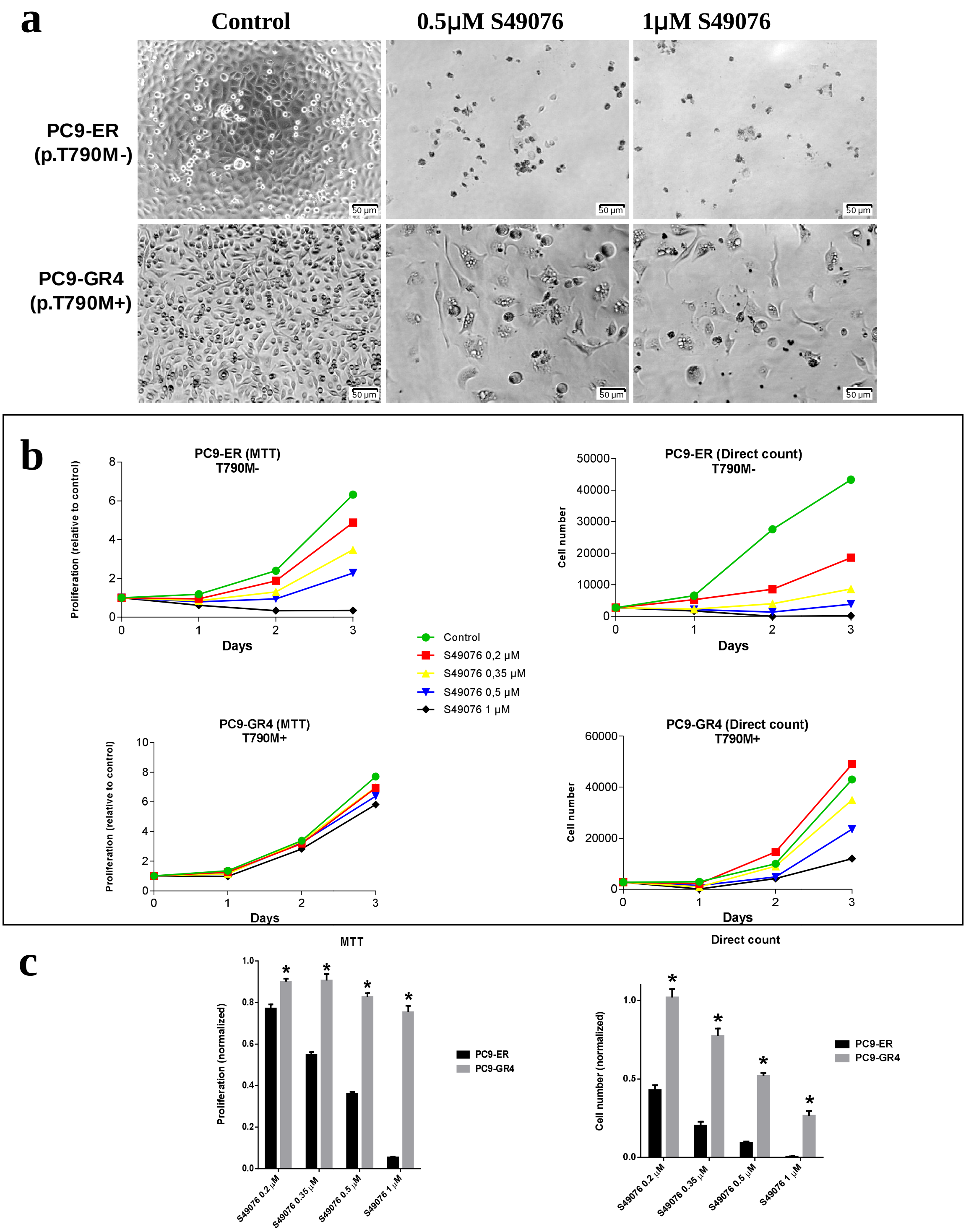

Supplementary Fig. 7: Effects of S49076 on PC9-ER (p.T790M-negative) and PC9-GR4 (p.T790M-positive) cell lines. **a** Microscopic photographs of the cells at different concentrations of the inhibitor. Scale bars indicate 50µm **b** Growth curves of the cell lines in presence of the inhibitors, determined by MTT (left) or direct counting (right). **c** Comparison of the final numbers of cells of the growth curves presented in (**b**), as estimated by MTT (left) or direct count (right). Values shown are means ± S.D. of six replicates and asterisks indicate statistical significance (*P*<0.05 in a Student's t test)

a

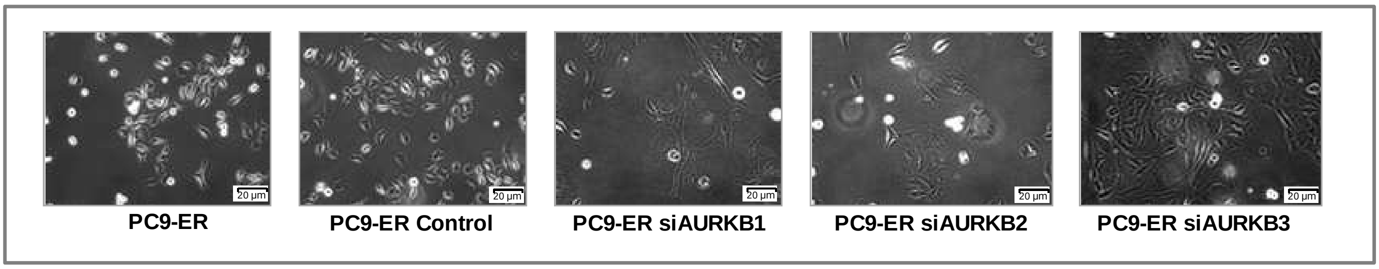

b

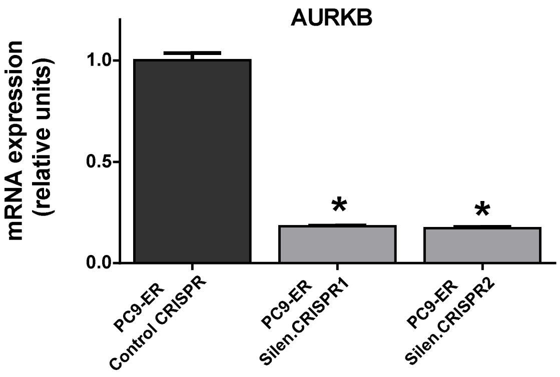

c

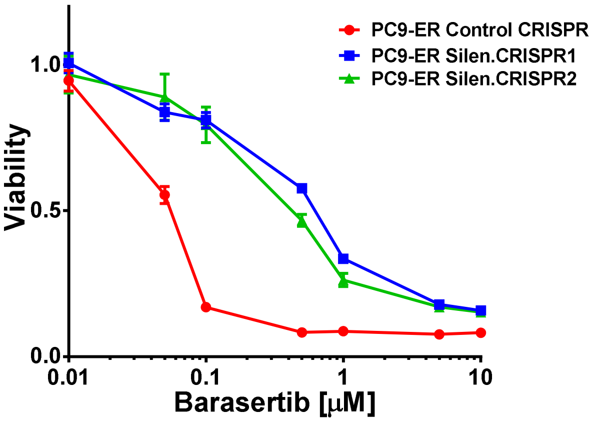

Supplementary Fig. 8: a Microscopic photographs of the parental PC9-ER cells, the clones partly silenced for *AURKB* (siAURKB1-3) and the clone transfected with a control plasmid. Scale bars indicate 20μm **b** Relative expression levels of the *AURKB* mRNAs in the PC9-ER control clone and the clones with AURKB CRISPR silencing**.** Values shown are means ± S.D. of three replicates and asterisks indicate statistical significance (P<0.05 in a Student's t test). **c** Dose-response plots to barasertib of the PC9-ER control clone and the clones with AURKB CRISPR silencing. Values shown are means ± S.D. of six replicates

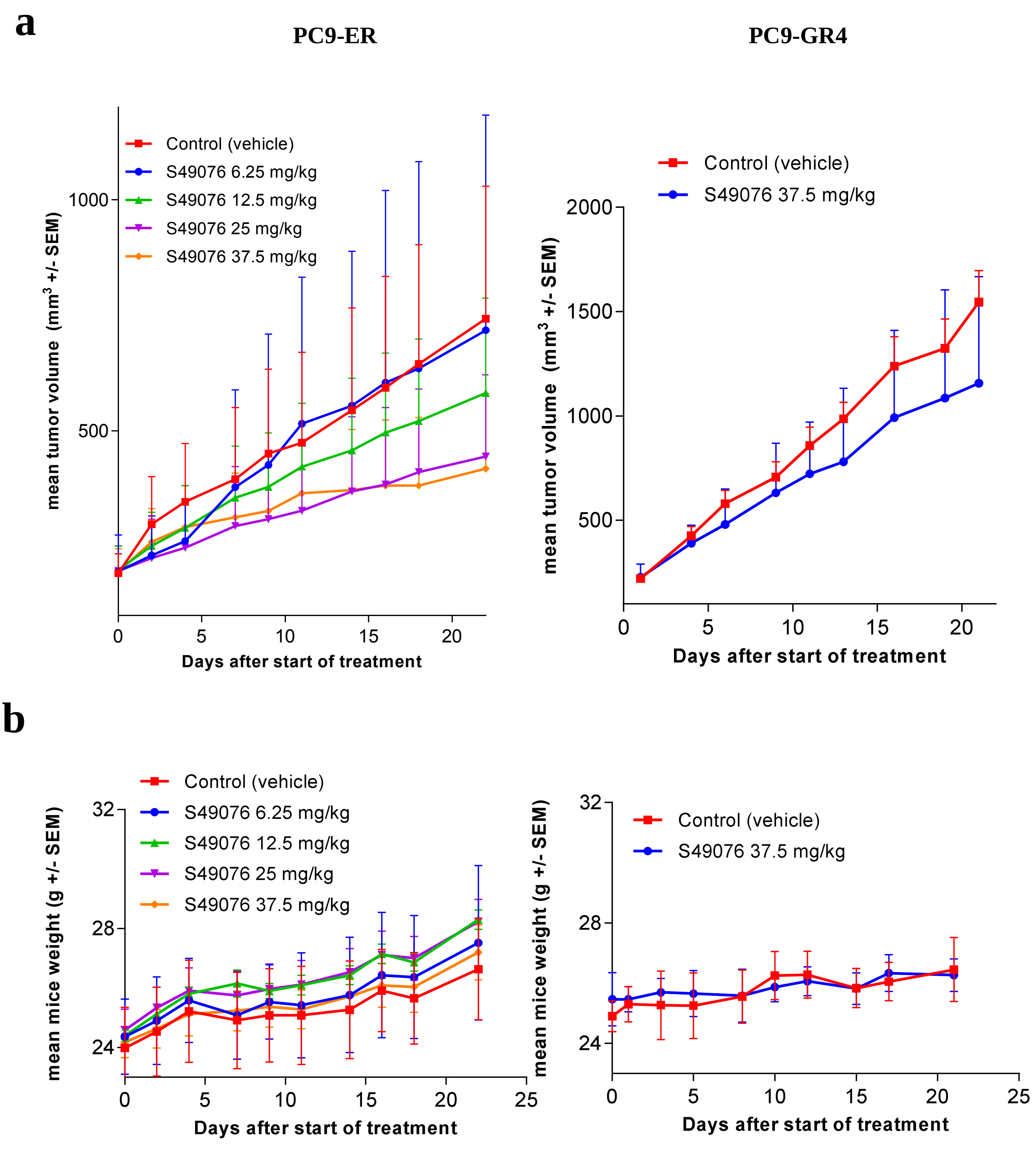

Supplementary Fig. 9: Effects of S49076 on the growth of subcutaneous PC9-ER (left panels) and PC9-GR4 xenografts (right panels). **a** Time course assessment of total tumor volume. **b** Weight of the animals used in the study. Values shown are means ± S.E.M of six replicates

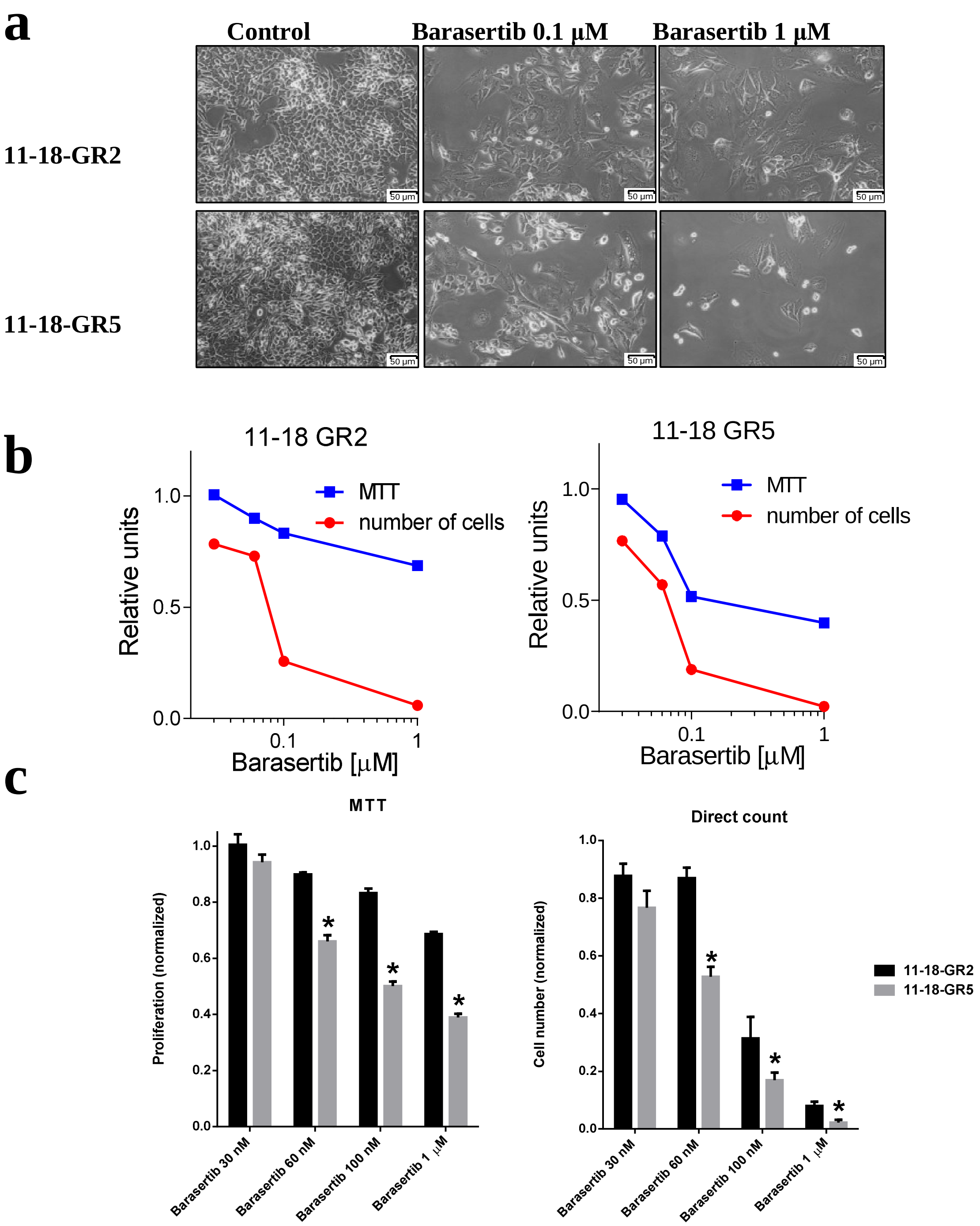

Supplementary Fig. 10: Effects of Barasertib on 11-18-GR5 (*NRAS* and T790M-negative) and 11-18-GR2 (*NRAS* Q61L) cell lines. **a** Microscopic photographs of the cells at different concentrations of the inhibitor. Scale bars indicate 50 µm**. b** Comparison of the dose-response to barasertib, as determined by MTT or direct counting in 11-18 GR2 (left) or 11-18 GR5 (right). **c** Comparison of the dose-response to barasertib of 11-18 GR2 and GR5, as determined by MTT (left) or direct counting (right). Values shown are means ± S.D. of six replicates and asterisks indicate statistical significance (P<0.05 in a Student's t test).

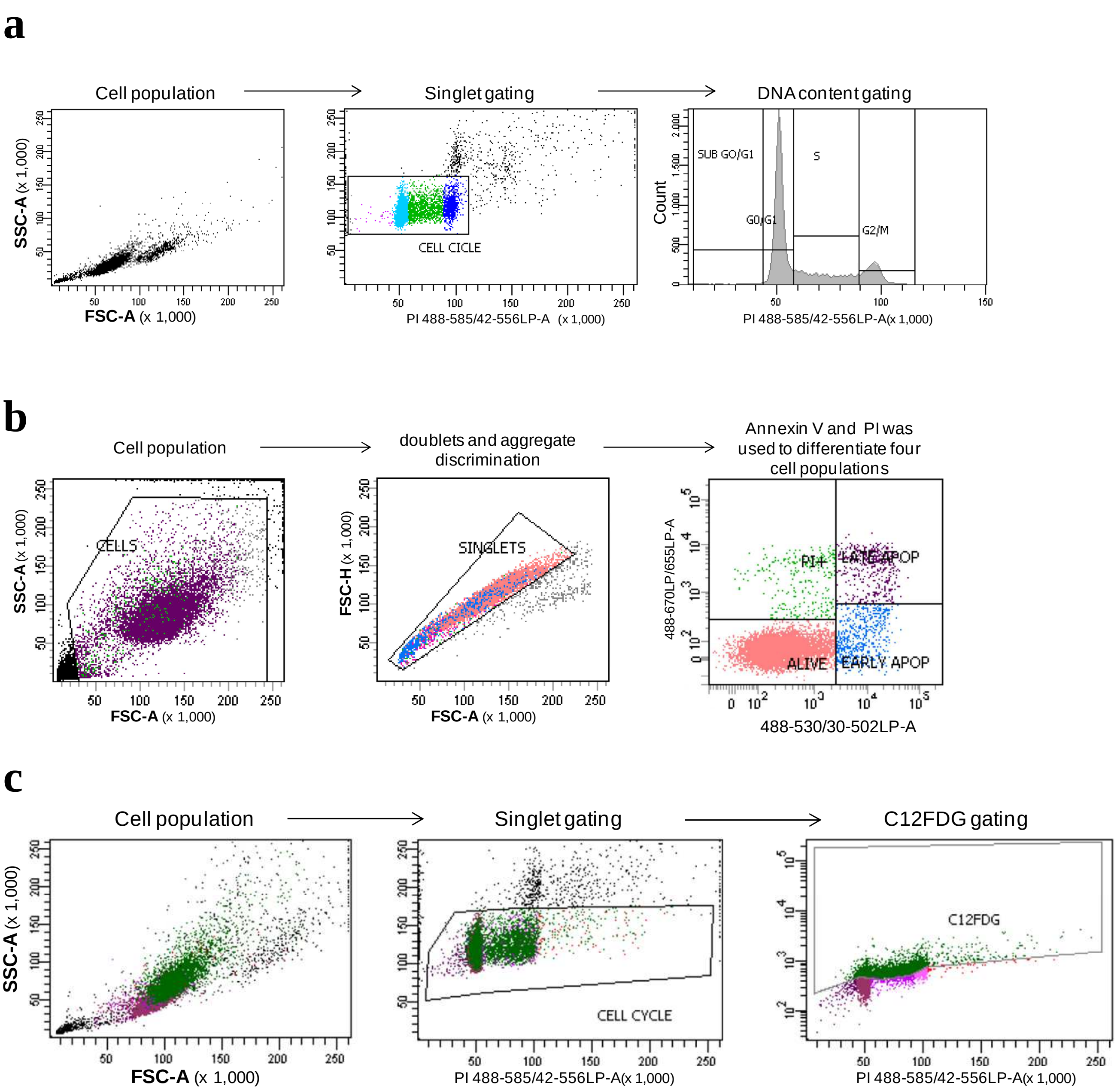

Supplementary Fig. 11: Representative FACS plots showing the gating strategy for flow cytometry analysis of: **a** cell-cycle profile (Propidium Iodide staining), **b** cell death (Annexin-V and Propidium Iodide staining), **c** senescent cells (C12FDG).

SUPPLEMENTARY TABLES

Supplementary Table 1: Doubling times and IC50 of selected inhibitors in the *AURKB* and *AXL* silenced clones derived from PC9-ER and used in this study. Values shown are the means of at least three different experiments. ND, not determined.

Cell line/Clone	Doubling time (h)	S49076	Barasertib	Tozasertib	BGB324	Foretinib	Crizotinib	Gefitinib
PC9-ER	27±2	0.3	0.06	0.2	2.3	0.7	1.4	12.2
PC9-ER siCont 1	27±4	0.3	0.04	0.2	2.1	0.6	1.6	10.4
PC9-ER siCont 2	31±3	0.4	0.05	0.2	2.3	0.6	1.8	8.8
PC9-ER siAXL1	31±7	0.5	0.05	ND	1.5	ND	1.7	10.6
PC9-ER siAXL2	31±2	0.4	0.05	ND	1.2	ND	1.6	10.3
PC9-ER siAXL3	ND	0.5	0.06	ND	1.0	ND	1.4	8.9
PC9-ER siAURKB1	28±3	>50	16.0	3.2	1.7	1.6	4.8	14.3
PC9-ER siAURKB2	30±4	>50	22.6	10.1	2.7	1.1	3.5	16.5
PC9-ER siAURKB3	28±4	>50	17.2	3.7	1.7	1.2	4.5	14.2

Supplementary Table 2: Characteristics of the 59 patients with *EGFR* mutant NSCLC treated with first line EGFR TKIs (pre-treatment samples) included in the study.

**Gender**	**no. (%)**
Male	15 (25.4)
Female	44 (74.6)
**Age - years**	
Median	66
Range	36−90
**ECOG performance status**	
0	15 (25.4)
1	26 (44.1)
2	15 (25.4)
3	3 (5.1)
**Smoking status**	
Never	41 (69.5)
Former	14 (23.7)
Current	4 (6.8)
**Disease stage**	
IIIB	14 (23.7)
IV	45 (76.3)
**Brain metastasis**	
No	40 (67.8)
Yes	19 (32.2)
**Bone metastasis**	
No	33 (55.9)
Yes	26 (44.1)
**Other metastatic sites**	
No	23 (39.0)
Yes	36 (61.0)
**Type of EGFR mutation**	
Exon 19 deletion	36 (61.0)
L858R	19 (32.2)
Other	4 (6.8)
**Type of EGFR TKI**	
Erlotinib	44 (58.7)
Gefitinib	29 (38.7)
Afatinib	2 (2.6)
**Response***	
PD	6 (10.1)
SD	19 (32.2)
PR	32 (54.2)
CR	2 (3.4)

*PD, progressive disease; SD, stable disease; PR, partial response; CR, complete response

Supplementary Table 3: Overall survival of *EGFR*-mut patients treated with EGFR TKIs (n=59) classified according to IHC H-scores.

**H-score**	**OS / Q4**	**OS / Q1-Q3**	**p (Long-rank)**
Non-mitotic pH3	18.8 months	34.9 months	0.413
Mitotic pH3	20.8 months	35.4 months	0.126
Ki67	21.9 months	36.7 months	0.071

Supplementary Table 4: Antibodies used in this study.

Antibody	Vendor (IHC)	Vendor (Western blotting)	Dilution
EGFR	--	Cell Signaling Technology (CST)-Cat#4267	1:1000
pEGFR	--	CST-Cat#3777	1:500
AXL	CST-Cat#8661	CST-Cat#8661	1:100 (IHC); 1:1000 (WB)
GAS6	R&D Systems-Cat# AF885	--	1:50
MET	Roche Diagnostics, Clone SP44-Cat#790-4430	CST-Cat#8198	Ready to use (IHC); 1:1000 (WB)
pMET	--	CST-Cat#3077	1:500
FGFR1	--	CST-Cat#9740	1:1000
FRS	--	R&D Systems-Cat#AF4069	1:1000
pFRS	--	CST-Cat#3861	1:500
ERK1/2	--	CST-Cat#9102	1:1000
pERK1/2	--	CST-Cat#9101	1:500
Akt	--	CST-Cat#9272	1:1000
pAkt	--	CST-Cat#9271	1:500
AURKB	Abcam-Cat# ab2254*	CST-Cat#3094	1:50 (ICC); 1:1000 (WB)
Ki67	Roche Diagnostics, Clone 30-9-Cat#790-4286	--	Ready to use
H3	--	Abcam-Cat# ab1791	1:1000
pH3	Abcam-Cat# ab5176	Millipore-Cat#06-570*	1:200 (IHC); 1:2000 (ICC); 1:250 (WB)
PARP		CST-Cat#9542	1:1000
Actin	--	Sigma Aldrich-Cat#A5441	1:2000
Tubulin	--	Sigma-Aldrich-Cat#T9026	1:2000

*Indicates the antibodies employed for ICC

Supplementary Table 5: Primers and probes used in this study

Gene		Sequence
AURKB	F	5´ CATGAGCCGCTCCAATGTC 3´
	R	5´ TGCTATTCTCCATCACCTTCTGG 3´
	Probe	6FAM 5´ AGCCCACAGCTGCC 3´ MGB
ATM	F	5´ TGGTGTGAAATATCTCAGCAACAGT 3´
	R	5´ CTTGTGAAGGTTTCAGATAGAGCCT 3´
	Probe	6FAM 5´ AGAATTGTTCTCTGTGTACTT 3´ MGB
NFKBIA	F	5´ TCCTCAACTTCCAGAACAACCTG 3´
	R	5´ TTCTGGCTGGTTGGTGATCA 3´
	Probe	6FAM 5´ CAGCAGACTCCACTCC 3´ MGB
53BP1	F	5´ GTCAGGTCATTGAGCAGTTACCTC 3´
	R	5´ TCCTCCACAGCAGGAGCAG 3´
	Probe	6FAM 5´ GGACAAGCAGTGTTCT 3´ MGB
CHEK2	F	5´TCCAAAGGCACGTTTTACGAC 3´
	R	5´TGTCTTCATCCTGAAGCCACG 3’
	Probe	6FAM 5´AGAAGAAGCCTTAAGACACC 3´ MGB
AXL silencing	shRNA	5´CCGGCGTGGAGAACAGCGAGATTTACTCGAGTAAA TCTCGCTGTTCTCCACGTTTTTTG 3´
AURBK silencing	shRNA	5´CCGGCCTGCGTCTCTACAACTATTTCTCGAGAAATA GTTGTAGAGACGCAGGTTTTT 3´
AURBK silencing (CRISPR)	sgRNA	5’ ATTCTAGAGTATGCCCCCCGCGG 3’
