# Supplementary material for: AURKB as a target in non-small cell lung cancer with acquired resistance to anti-EGFR therapy
Source: Nat Commun. 2019 Apr 18;10:1812. doi: 10.1038/s41467-019-09734-5 (PMC6472415; doi:10.1038/s41467-019-09734-5)
Supplement: Supplementary file 3 — Reporting Summary [file 41467_2019_9734_MOESM3_ESM.pdf]

nature research

Corresponding author(s): Miguel A Molina-Vila

Reporting Summary

Nature Research wishes to improve the reproducibility of the work that we publish. This form provides structure for consistency and transparency in reporting. For further information on Nature Research policies, see Authors & Referees and the Editorial Policy Checklist.

Statistical parameters

When statistical analyses are reported, confirm that the following items are present in the relevant location (e.g. figure legend, table legend, main text, or Methods section).

n/a	Confirmed	
☐	☒	The exact sample size (*n*) for each experimental group/condition, given as a discrete number and unit of measurement
☐	☒	An indication of whether measurements were taken from distinct samples or whether the same sample was measured repeatedly
☐	☒	The statistical test(s) used AND whether they are one- or two-sided<br>*Only common tests should be described solely by name; describe more complex techniques in the Methods section.*
☒	☐	A description of all covariates tested
☒	☐	A description of any assumptions or corrections, such as tests of normality and adjustment for multiple comparisons
☐	☒	A full description of the statistics including central tendency (e.g. means) or other basic estimates (e.g. regression coefficient) AND variation (e.g. standard deviation) or associated estimates of uncertainty (e.g. confidence intervals)
☐	☒	For null hypothesis testing, the test statistic (e.g. *F*, *t*, *r*) with confidence intervals, effect sizes, degrees of freedom and *P* value noted<br>*Give P values as exact values whenever suitable.*
☒	☐	For Bayesian analysis, information on the choice of priors and Markov chain Monte Carlo settings
☒	☐	For hierarchical and complex designs, identification of the appropriate level for tests and full reporting of outcomes
☒	☐	Estimates of effect sizes (e.g. Cohen's *d*, Pearson's *r*), indicating how they were calculated
☐	☒	Clearly defined error bars<br>*State explicitly what error bars represent (e.g. SD, SE, CI)*

Our web collection on statistics for biologists may be useful.

Software and code

Policy information about availability of computer code

Data collection	Primer and probe sets were designed using Primer Express 3.0 Software<br>Western blot images were collected with the chemidoc mp imaging system<br>96-well plates were read using the Tecan i-control 2.0.10.0 software
Data analysis	Flow cytometry data was analyzed using the FACSDiva software version 6.1.2.<br>Statistical analyses were performed using the GraphPad Prism v6.0 Software.

For manuscripts utilizing custom algorithms or software that are central to the research but not yet described in published literature, software must be made available to editors/reviewers upon request. We strongly encourage code deposition in a community repository (e.g. GitHub). See the Nature Research guidelines for submitting code & software for further information.

Data

Policy information about availability of data

All manuscripts must include a data availability statement. This statement should provide the following information, where applicable:
- Accession codes, unique identifiers, or web links for publicly available datasets
- A list of figures that have associated raw data
- A description of any restrictions on data availability

The NGS data generated in this study have been deposited in the in the Sequence Read Archive (SAR) of the National Center for Biotechnology Information (NCBI), under the accession codes PRJNA524804 (project) and SAMN11035311-SAMN11035324 (individual samples).. The source data underlying Figs 1e, 2a, 2g, 3c, 3e-g, 4a, 4b, 4e, 4f, 4h, 5c-e, 6a, 6c and Supplementary Figs 1b, 2, 3c, 3d, 4a, 4b, 5b, 5c, 6a, 6c, 7b, 7c, 9a, 9b, 10b, 10c are provided as a source data file. The file also contains uncropped and unprocessed scans of the Western blots presented in Figs 1b, 2c, 2d and 3a. The source data file has also been deposited in the Open Science Framework (OSF) repository under the unique identifier DOI 10.17605/OSF.IO/JW4C7. The authors declare that all other data supporting the findings of this study are available within the main article and its Supplementary Information file or available from corresponding authors upon reasonable request. A reporting summary for this article is available as Supplementary Information file.

Field-specific reporting

Please select the best fit for your research. If you are not sure, read the appropriate sections before making your selection.

☒ Life sciences ☐ Behavioural & social sciences ☐ Ecological, evolutionary & environmental sciences

For a reference copy of the document with all sections, see nature.com/authors/policies/ReportingSummary-flat.pdf

Life sciences study design

All studies must disclose on these points even when the disclosure is negative.

Sample size	For proliferation experiments and IC50 calculations, the sample size (number of replicate wells per experiment) was 6. This a standard number, derived from the fact that proliferation experiments are performed in 8x12 well plates. Since the exterior wells are not used, there are 6 available wells per column.<br>In the case of the mice experiments, we selected 6 animals per group based on our previous experience in this kind of studies<br>Finally, for the analysis of pH3 in human samples, we selected all the biopsies of EGFR-mut NSCLC lung cancer patients available at our institution (68 pretreatment, 24 rebiopsies after relapse)
Data exclusions	The data from one xenograft tumor was excluded in Fig. 3f. It was identified using the "outlier" tool of the GraphPad Prism 6.01 program
Replication	In order to verify the reproducibility of the experimental findings, most experiments in cultured cell lines were repeated at least three times. In addition, 19 cell line models were used to verify the reproducibility of the main finding of the manuscript (apoptosis vs. senescence in response to AURKB inhibition in NSCLC cells resistant to EGFR TKIs)
Randomization	In the tumor xenograft studies all animals were weighted and tumor volumes were measured before starting the treatment. Since initial tumor volume can affect the effectiveness of any given treatment, mice were assigned into groups using randomized block design based upon their tumor volumes (see page 25 of the manuscript). During randomization outliers were discarded. This is a precaution to ensure that all groups are comparable at baseline.
Blinding	The investigators were blinded for group allocation and data collection in the animal studies, flow cytometry experiments and immunohistochemistry evaluations. Blinding was not relevant in the rest of experiments, since measures (absorbances or bands in a gel) are taken automatically with no human intervention

Reporting for specific materials, systems and methods

Materials & experimental systems

n/a	Involved in the study
☐	☒ Unique biological materials
☐	☒ Antibodies
☐	☒ Eukaryotic cell lines
☐	☐ Palaeontology
☐	☒ Animals and other organisms
☐	☒ Human research participants

Methods

n/a	Involved in the study
☐	☐ ChIP-seq
☐	☒ Flow cytometry
☐	☐ MRI-based neuroimaging

Unique biological materials

Policy information about availability of materials

Obtaining unique materials	All unique materials (resistant cell lines) are readily available from the authors

Antibodies

Antibodies used	Antibodies used are described in the Supplementary Table 4 of the manuscript
Validation	All antibodies used have been validated by the vendors

Eukaryotic cell lines

Policy information about cell lines

Cell line source(s)	The H1975 cell line was purchased from the ATCC. Parental PC9 cells were provided by F. Hoffman-La Roche Ltd (Basel, Switzerland) with the authorization of Dr. Mayumi Ono (Kyushu University, Fukuoka, Japan). Parental 11–18 cells were kindly provided by Dr. Mayumi Ono. Resistant cells were derived by the authors by culturing parental cells with EGFR TKIs (page 21 of the manuscript)
Authentication	Cell lines were authenticated by analyzing >20 polymorphisms by NGS. In all cases, the genotypes and allelic fractions of parental and resistant cells were identical (page 21)
Mycoplasma contamination	All cells were routinely tested for mycoplasma contamination and were consistently negative
Commonly misidentified lines (See ICLAC register)	No commonly misidentified lines were used

Palaeontology

Specimen provenance	*Provide provenance information for specimens and describe permits that were obtained for the work (including the name of the issuing authority, the date of issue, and any identifying information).*
Specimen deposition	*Indicate where the specimens have been deposited to permit free access by other researchers.*
Dating methods	*If new dates are provided, describe how they were obtained (e.g. collection, storage, sample pretreatment and measurement), where they were obtained (i.e. lab name), the calibration program and the protocol for quality assurance OR state that no new dates are provided.*

☐ Tick this box to confirm that the raw and calibrated dates are available in the paper or in Supplementary Information.

Animals and other organisms

Policy information about studies involving animals; ARRIVE guidelines recommended for reporting animal research

Laboratory animals	The mice used in the study had the following characteristics<br>Species: Mus musculus<br>Strain: BalbC<br>Age: 5 weeks<br>Sex: Male<br>Body weight at reception: 20-22 g
Wild animals	The study did not involve wild animals
Field-collected samples	The study did not involve samples collected from the field

Human research participants

Policy information about studies involving human research participants

Population characteristics	In the study we used pre-treatment biopsy samples from 59 patients with EGFR mutant NSCLC treated with first line EGFR TKIs. The characteristics of the patients can be found in the Supplementary Table 2 of the manuscript
Recruitment	All EGFR-mut NSCLC patients with biopsies available, diagnosed between 2006 and 2017 in the Dexeus Quirón University Hospital (Barcelona, Spain) and Fundación Santa Fe de Bogotá (Colombia) and treated in these institutions were included in the study

ChIP-seq

Data deposition

☐ Confirm that both raw and final processed data have been deposited in a public database such as GEO.

☐ Confirm that you have deposited or provided access to graph files (e.g. BED files) for the called peaks.

Data access links<br>*May remain private before publication.*	*For "Initial submission" or "Revised version" documents, provide reviewer access links. For your "Final submission" document, provide a link to the deposited data.*
Files in database submission	*Provide a list of all files available in the database submission.*
Genome browser session<br>(e.g. UCSC)	*Provide a link to an anonymized genome browser session for "Initial submission" and "Revised version" documents only, to enable peer review. Write "no longer applicable" for "Final submission" documents.*

Methodology

Replicates	*Describe the experimental replicates, specifying number, type and replicate agreement.*
Sequencing depth	*Describe the sequencing depth for each experiment, providing the total number of reads, uniquely mapped reads, length of reads and whether they were paired- or single-end.*
Antibodies	*Describe the antibodies used for the ChIP-seq experiments; as applicable, provide supplier name, catalog number, clone name, and lot number.*
Peak calling parameters	*Specify the command line program and parameters used for read mapping and peak calling, including the ChIP, control and index files used.*
Data quality	*Describe the methods used to ensure data quality in full detail, including how many peaks are at FDR 5% and above 5-fold enrichment.*
Software	*Describe the software used to collect and analyze the ChIP-seq data. For custom code that has been deposited into a community repository, provide accession details.*

Flow Cytometry

Plots

Confirm that:

☒ The axis labels state the marker and fluorochrome used (e.g. CD4-FITC).

☒ The axis scales are clearly visible. Include numbers along axes only for bottom left plot of group (a 'group' is an analysis of identical markers).

☒ All plots are contour plots with outliers or pseudocolor plots.

☒ A numerical value for number of cells or percentage (with statistics) is provided.

Methodology

Sample preparation	Senescent:<br>Cells (3 × 104) were grown in T-25 flasks and treated with selected drugs and incubated for 1 h with fresh RPMI+10%FBS with 100 nM bafilomycin A1 to neutralize the acidic pH of lysosomes. Finally, 5-dodecanoylaminofluorescein di-β-D-galactopyranoside (C12FDG) was added at a final concentration of 33 µM for 2 h and cells were submitted to flow cytometry.<br>Annexin:<br>Wash 1x106 cells with PBS and centrifuge cells at 200 x g for 5 min.<br>Resuspend the cell pellet in 100uL of Annexin-V-FLUOS labeling solution. Incubate 10-15 min at 15-25ºC.
Instrument	BD FACSCanto™ II system.
Software	FACSDiva Version 6.1.2.
Cell population abundance	Flow cytometry was used for quantification purposes only (i.e. no postsorting fractions were collected).
Gating strategy	Gating strategy is presented in Supplementary Fig.11.

☒ Tick this box to confirm that a figure exemplifying the gating strategy is provided in the Supplementary Information.

Magnetic resonance imaging

Experimental design

Design type — *Indicate task or resting state; event-related or block design.*

Design specifications — *Specify the number of blocks, trials or experimental units per session and/or subject, and specify the length of each trial or block (if trials are blocked) and interval between trials.*

Behavioral performance measures — *State number and/or type of variables recorded (e.g. correct button press, response time) and what statistics were used to establish that the subjects were performing the task as expected (e.g. mean, range, and/or standard deviation across subjects).*

Acquisition

Imaging type(s) — *Specify: functional, structural, diffusion, perfusion.*

Field strength — *Specify in Tesla*

Sequence & imaging parameters — *Specify the pulse sequence type (gradient echo, spin echo, etc.), imaging type (EPI, spiral, etc.), field of view, matrix size, slice thickness, orientation and TE/TR/flip angle.*

Area of acquisition — *State whether a whole brain scan was used OR define the area of acquisition, describing how the region was determined.*

Diffusion MRI ☐ Used ☐ Not used

Preprocessing

Preprocessing software — *Provide detail on software version and revision number and on specific parameters (model/functions, brain extraction, segmentation, smoothing kernel size, etc.).*

Normalization — *If data were normalized/standardized, describe the approach(es): specify linear or non-linear and define image types used for transformation OR indicate that data were not normalized and explain rationale for lack of normalization.*

Normalization template — *Describe the template used for normalization/transformation, specifying subject space or group standardized space (e.g. original Talairach, MNI305, ICBM152) OR indicate that the data were not normalized.*

Noise and artifact removal — *Describe your procedure(s) for artifact and structured noise removal, specifying motion parameters, tissue signals and physiological signals (heart rate, respiration).*

Volume censoring — *Define your software and/or method and criteria for volume censoring, and state the extent of such censoring.*

Statistical modeling & inference

Model type and settings — *Specify type (mass univariate, multivariate, RSA, predictive, etc.) and describe essential details of the model at the first and second levels (e.g. fixed, random or mixed effects; drift or auto-correlation).*

Effect(s) tested — *Define precise effect in terms of the task or stimulus conditions instead of psychological concepts and indicate whether ANOVA or factorial designs were used.*

Specify type of analysis: ☐ Whole brain ☐ ROI-based ☐ Both

Statistic type for inference (See Eklund et al. 2016) — *Specify voxel-wise or cluster-wise and report all relevant parameters for cluster-wise methods.*

Correction — *Describe the type of correction and how it is obtained for multiple comparisons (e.g. FWE, FDR, permutation or Monte Carlo).*

Models & analysis

n/a	Involved in the study
☐	☐ Functional and/or effective connectivity
☐	☐ Graph analysis
☐	☐ Multivariate modeling or predictive analysis

Functional and/or effective connectivity — *Report the measures of dependence used and the model details (e.g. Pearson correlation, partial correlation, mutual information).*

Graph analysis — *Report the dependent variable and connectivity measure, specifying weighted graph or binarized graph, subject- or group-level, and the global and/or node summaries used (e.g. clustering coefficient, efficiency, etc.).*

Multivariate modeling and predictive analysis

Specify independent variables, features extraction and dimension reduction, model, training and evaluation metrics.
